# Supplementary material for: The retinal ipRGC-preoptic circuit mediates the acute effect of light on sleep
Source: Nat Commun. 2021 Aug 25;12:5115. doi: 10.1038/s41467-021-25378-w (PMC8387462; doi:10.1038/s41467-021-25378-w)
Supplement: Supplementary file 1 — Supplementary Information [file 41467_2021_25378_MOESM1_ESM.pdf]

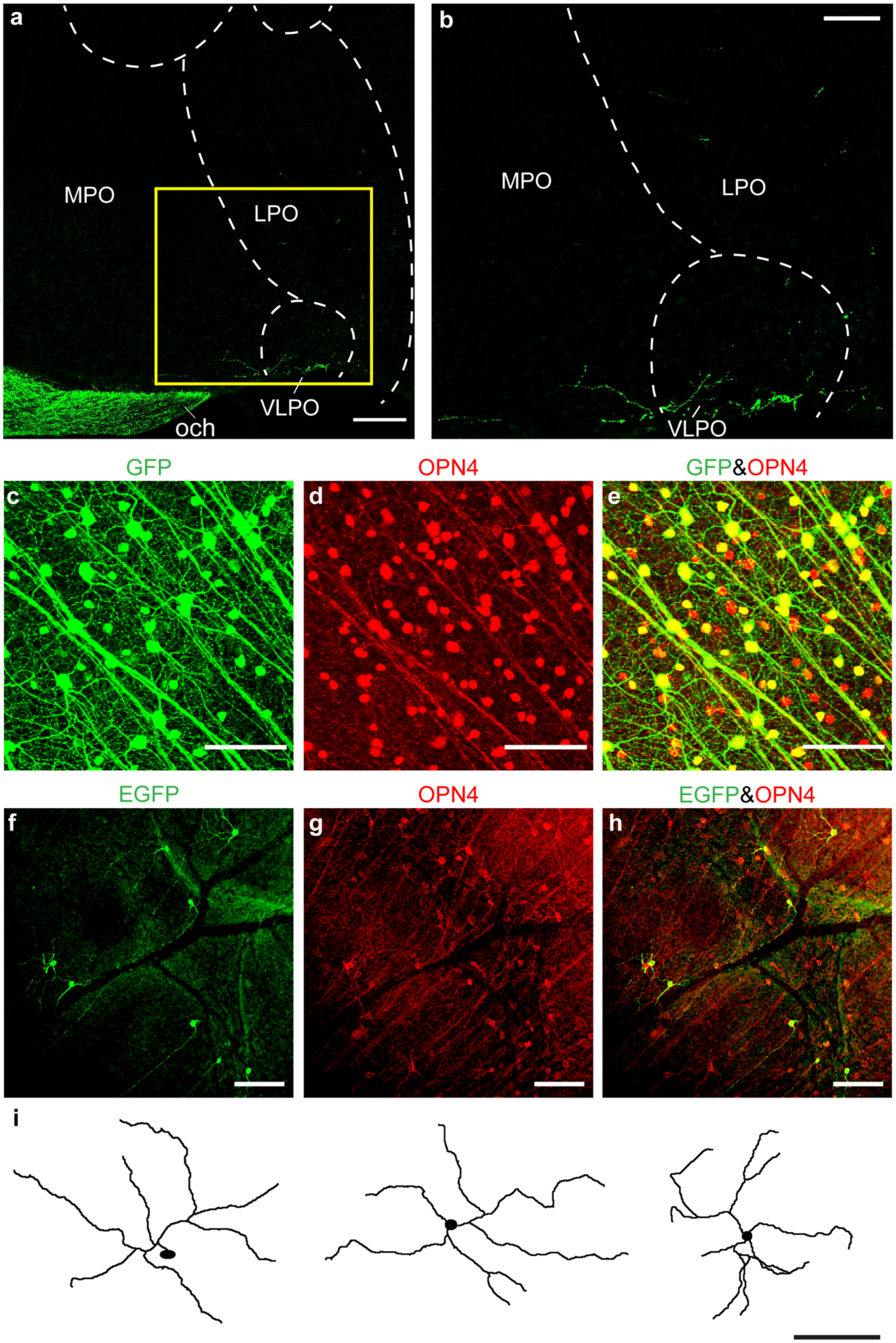

a
MPO
LPO
VLPO
och
b
MPO
LPO
VLPO
GFP
OPN4
GFP&OPN4
c
d
e
EGFP
OPN4
EGFP&OPN4
f
g
h
i

Supplementary Figure 1 M1 ipRGCs are the predominant subtype that projects to the POA and ipRGCs project beyond the VLPO to the MPO and the LPO. **a,** Immunostaining of GFP in the MPO, the LPO, and the VLPO in *Opn4*^{Cre} mice that are injected with AAV2-CAG-DIO-GFP virus in the eye (n = 10 retinas from 5 animals; scale bar, 200 μm). The boxed region in **a** is enlarged in **b** showing fibers outside the VLPO (n = 10 retinas from 5 animals; scale bar, 100 μm). **c-e,** Distribution of GFP antibody-positive cells (**c**), OPN4 mCherry-expressing cells (**d**), and colocalization (**e**) in the retina of *Opn4*^{Cre}*;Ai9* mice that are injected with AAV2-CAG-DIO-GFP virus in the eye (n = 4 retinas from 2 animals; scale bar, 100 μm). **f-h,** Distributions of EGFP positive cells (**f**), OPN4 antibody-positive cells (**g**), and colocalization (**h**) in the retina of *Opn4*^{Cre} mice that are injected with AAV2-Ef1α-DIO-Flp virus in the eye and AAVretro-Ef1α-fDIO-hM3D(Gq)-EGFP virus in the POA (n = 4 retinas from 2 animals; scale bar, 100 μm). **i,** Retinal reconstruction of ipRGCs that project to the POA. All three reconstructed cells belong to the M1 ipRGC subtype (Scale bar, 100 μm). MPO, medial preoptic area; LPO, lateral preoptic area; VLPO, ventrolateral preoptic nucleus; och, optic chiasm.

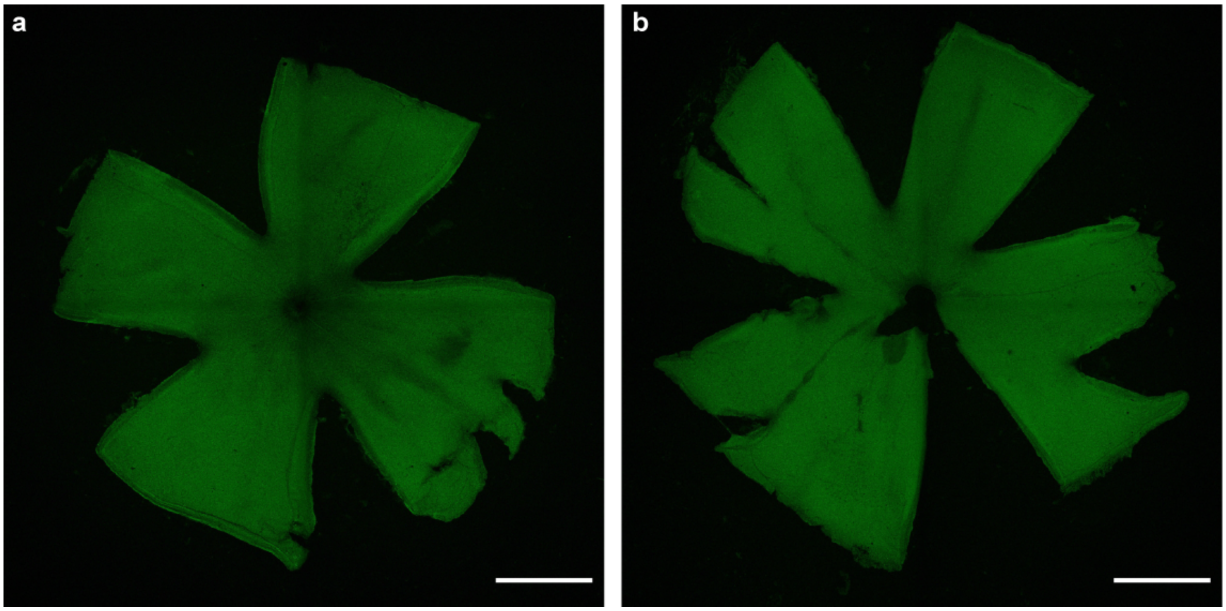

Supplementary Figure 2 Viral expression depends on Cre. **a, b,** No GFP expression in the retina of wild type mouse that was injected AAV2-CAG-DIO-GFP in the eye **(a)** (n = 4 retinas from 2 animals; scale bar, 1000 μm); or that was injected AAV2-Ef1α-DIO-Flp in the eye and AAVretro-Ef1α-fDIO-hM3D(Gq)-EGFP in the POA **(b)** (n = 6 retinas from 3 animals; scale bar, 1000 μm).

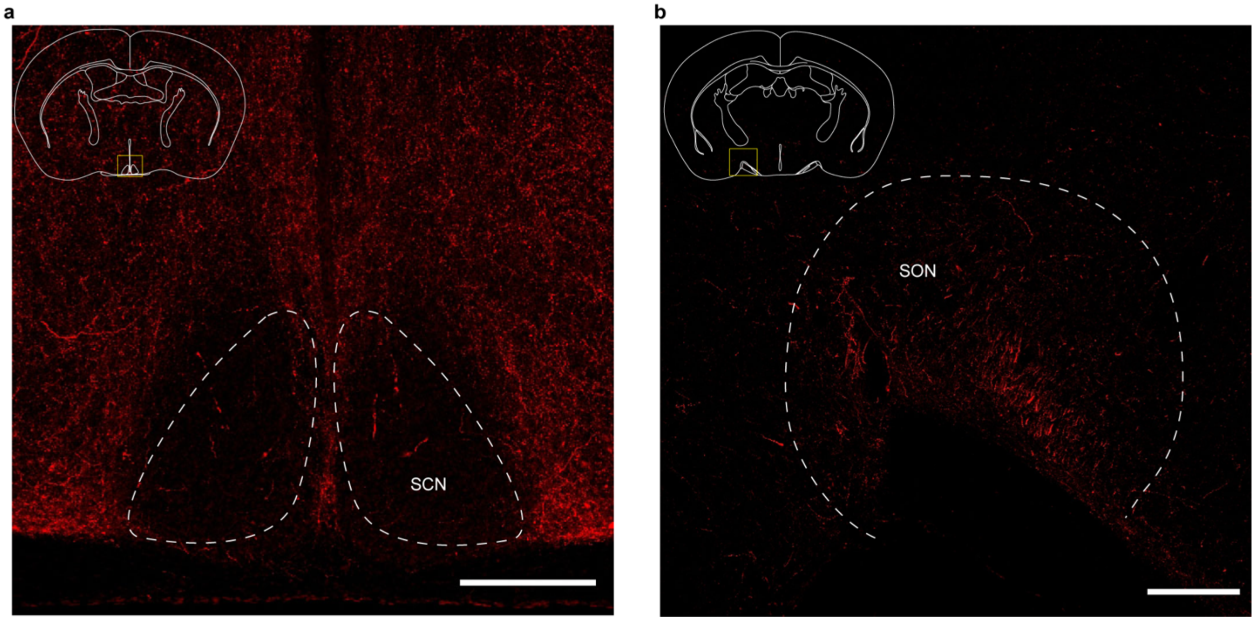

Supplementary Figure 3 The SCN and the SON did not show mCherry expression after viral infection. **a, b,** AAV8-hSyn-mCherry injection in the POA of *Opn4*^{Cre} mice did not show expression in the somas of the SCN (**a**), or the SON (**b**). The fibers in the SCN and SON arise from the injection of non-Cre dependent virus expressing mCherry in the POA. n = 8 animals. Scale bars denote 200 µm. SCN, suprachiasmatic nucleus; SON, supraoptic nucleus; POA, preoptic area.

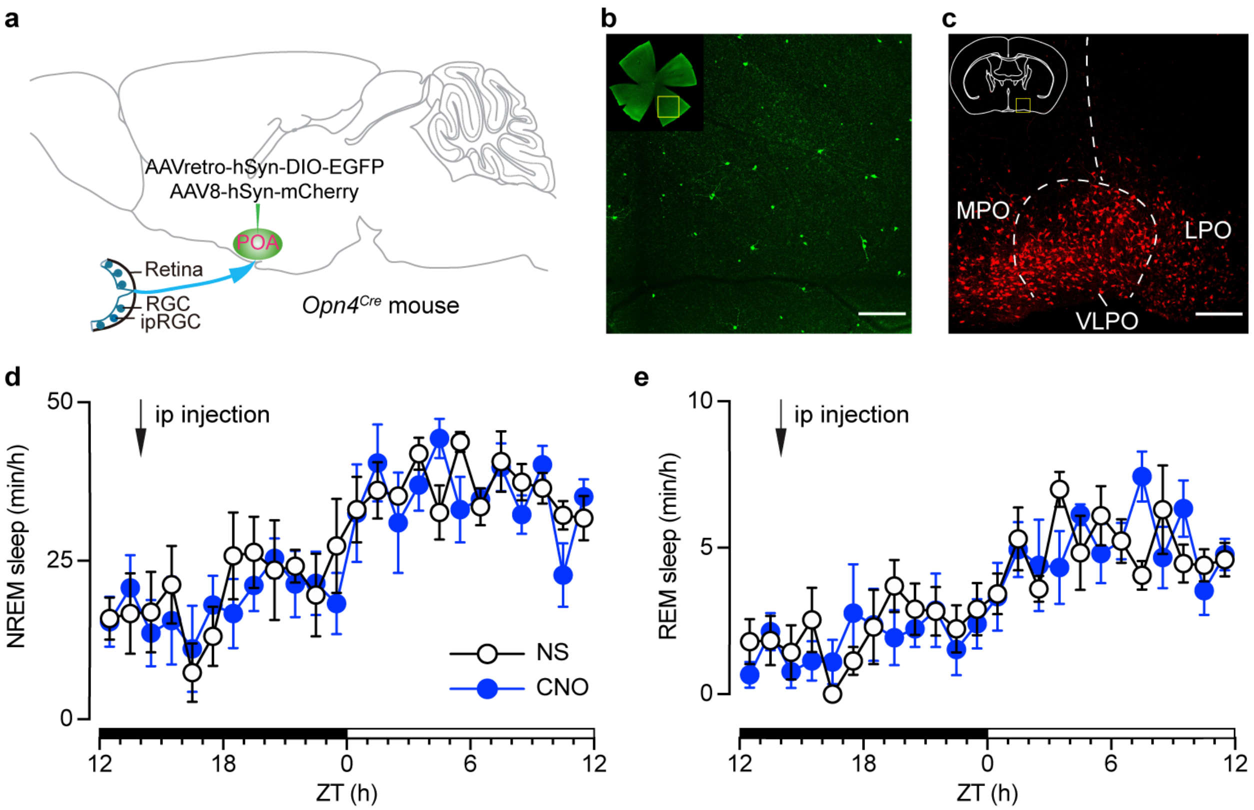

Supplementary Figure 4 CNO injection did not affect NREM or REM sleep in *Opn4*^{Cre} mice injected with AAVretro-hSyn-DIO-EGFP, that expresses only EGFP in the retina. **a,** Schematic of the viral strategy employed. **b,** Immunostaining of EGFP in the retina shows labeling of ipRGCs with EGF (n = 10 retinas from 5 animals; scale bar, 200 μm). **c,** Immunostaining of mCherry in the POA to delineate the injection site (n = 5 animals; scale bar, 200 μm). **d,** Time course changes in NREM sleep after NS or CNO injection (n = 5 animals; two-way ANOVA, $F_{1, 8}$ = 1.508, P = 0.2544, Bonferroni post hoc test). The black and white bars on the x axes indicate light-off and light-on periods, respectively. e, Time course changes in REM sleep after NS or CNO injection (n = 5 animals; two-way ANOVA, $F_{1, 8}$ = 0.5040, P = 0.4979, Bonferroni post hoc test). All error bars denote SEM. ipRGC, intrinsically photosensitive retinal ganglion cell; MPO, medial preoptic area; LPO, lateral preoptic area; VLPO, ventrolateral preoptic nucleus; NREM, non-rapid eye movement.

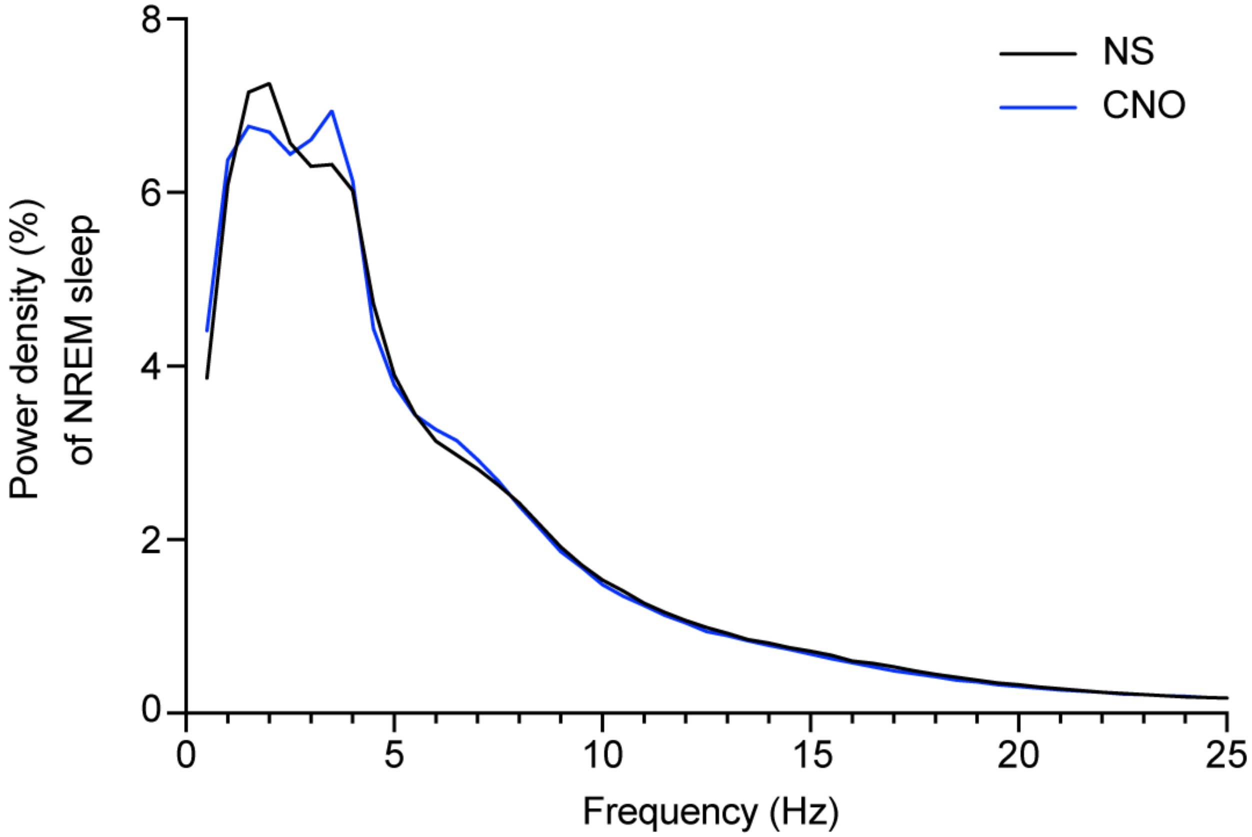

Supplementary Figure 5 Activation of POA light-responsive neurons did not change the power density of NREM sleep. Although NREM sleep was increased, the power density was not affected between the normal saline (NS) and clozapine-N-oxide (CNO) treated animals. n = 5 animals. NREM, non-rapid eye movement.

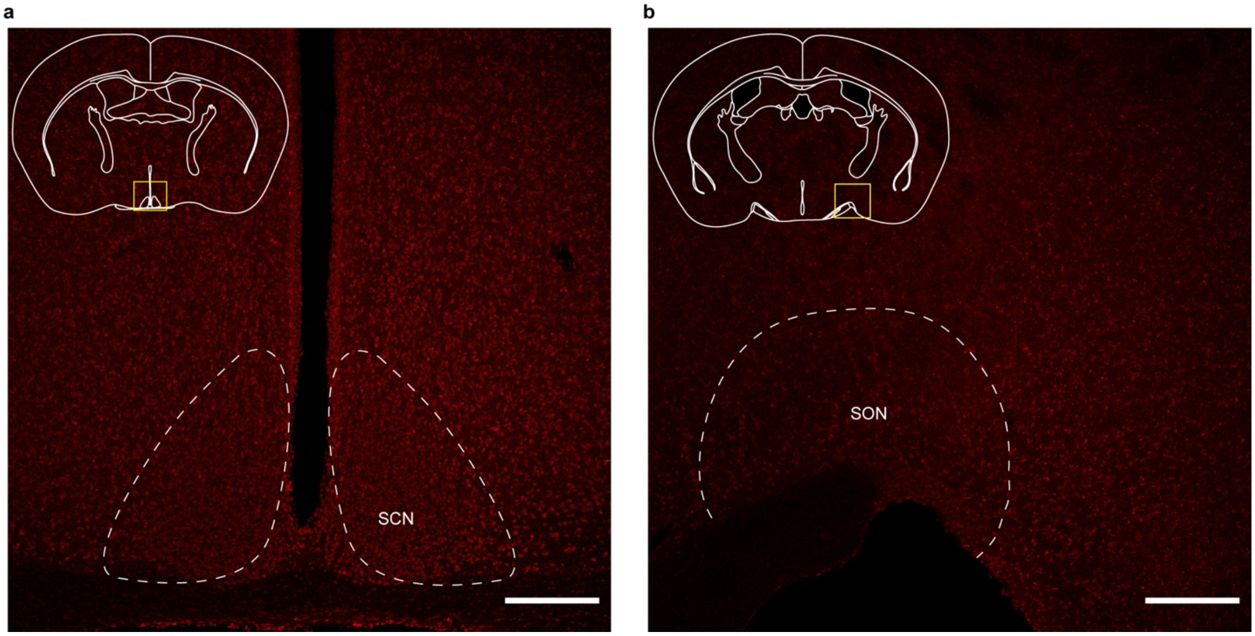

Supplementary Figure 6 The SCN and SON did not show mCherry expression after viral infection in Fos-2A-iCreER mice. **a, b,** AAV8-hSyn-DIO-hM3D(Gq)-mCherry injected in the POA of Fos-2A-iCreER mice treated with light and 4-Hydroxytamoxifen did not show mCherry expression in somas of the SCN (**a**), or the SON (**b**). Note the absence of fiber innervation from the activated light-responsive POA neurons in comparison to Supplementary Figure 3. n = 5 animals. Scale bars denote 200 μm. SCN, suprachiasmatic nucleus; SON, supraoptic nucleus; POA, preoptic area.

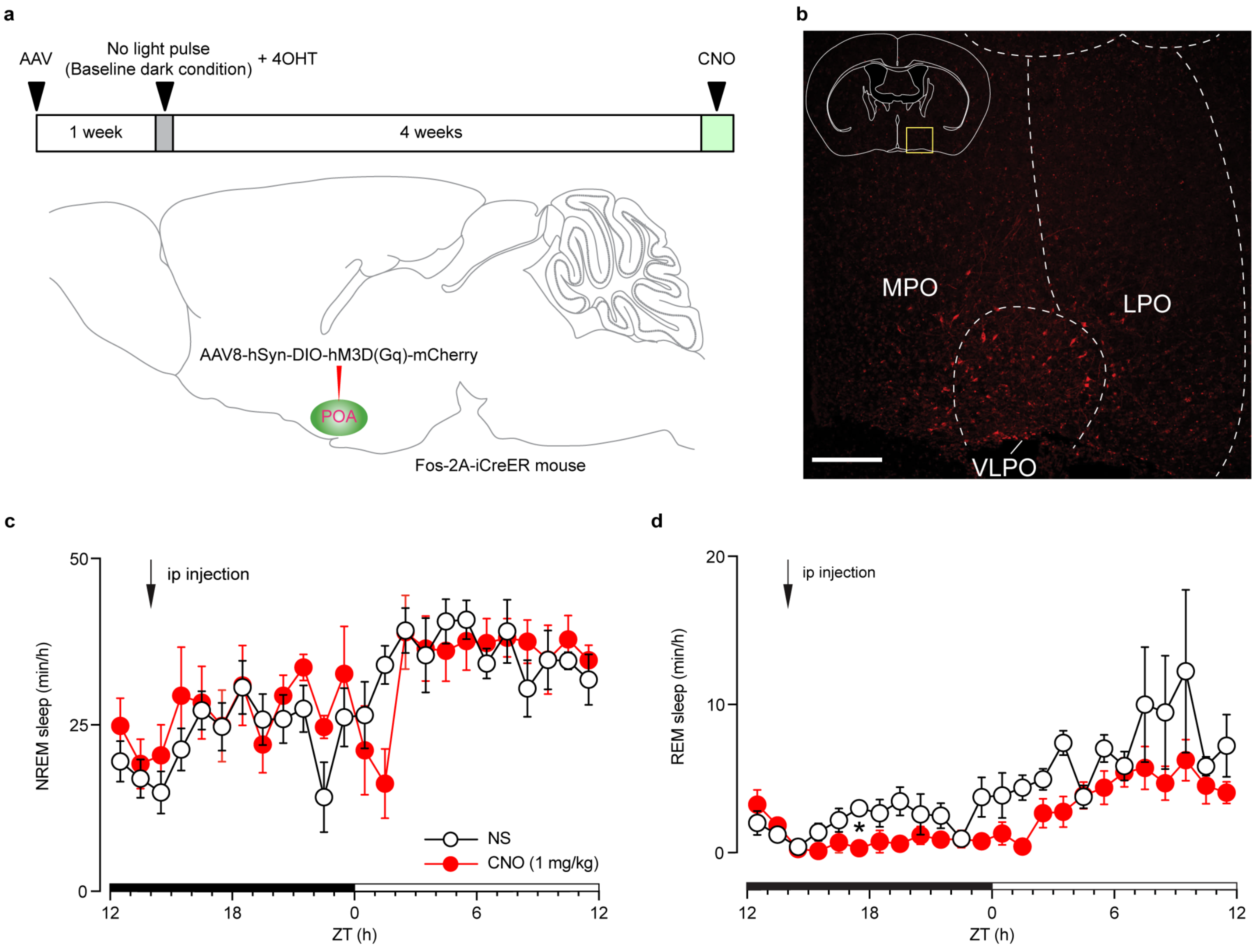

Supplementary Figure 7 The effect of activation of night-active POA neurons on sleep. **a,** Schematic showing viral, 4-Hydroxytamoxifen (4-OHT) and CNO injections in Fos-2A-iCreER mice. **b,** 4-OHT induced the expression of mCherry in the POA (n = 5 animals; scale bar, 200 μm). **c,** Time course changes in NREM sleep after normal saline (NS) or clozapine-N-oxide (CNO) injection (n = 5 animals; two-way ANOVA, $F_{1, 10} = 1.426$, P = 0.2599, Bonferroni post hoc test). Each cycle represents the hourly mean ± SEM of sleep. The black and white bars on the x axes indicate light-off and light-on periods, respectively. **d,** Time course changes in REM sleep after NS or CNO injection (n = 5 animals; two-way ANOVA, $F_{1, 10} = 5.697$, P = 0.0382, Bonferroni post hoc test). All error bars denote SEM. MPO, medial preoptic area; LPO, lateral preoptic area; VLPO, ventrolateral preoptic nucleus; ZT, zeitgeber time.

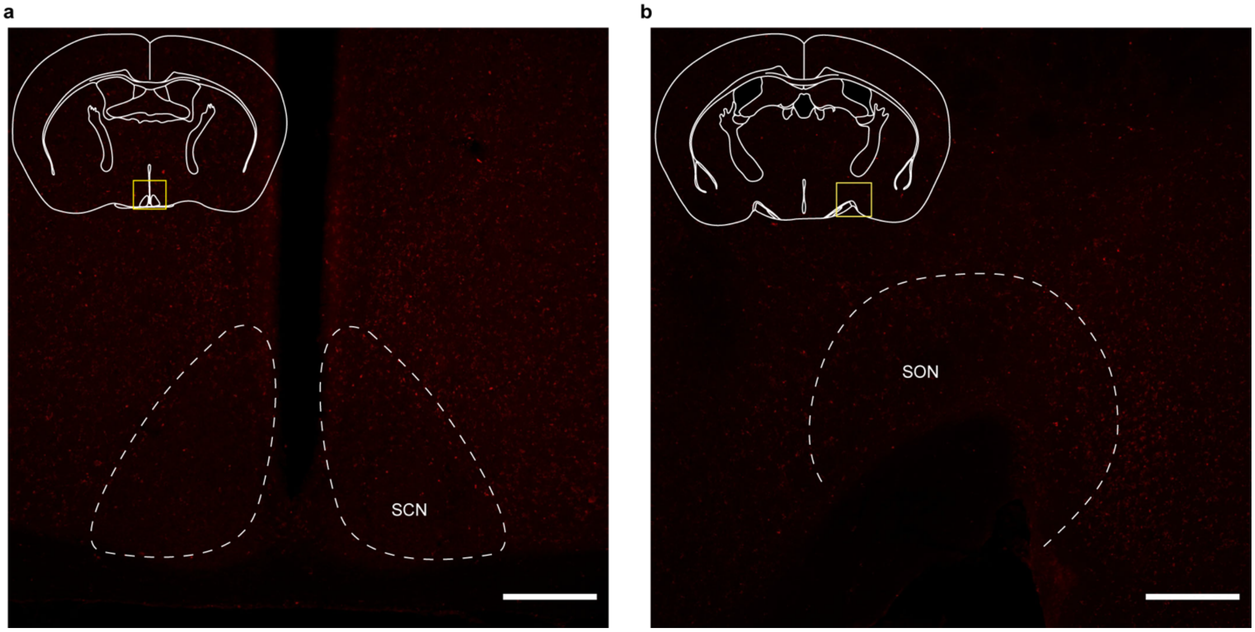

Supplementary Figure 8 The SCN and SON did not show mCherry expression after viral infection in Fos-2A-iCreER mice. **a, b,** AAV8-hSyn-DIO-hM4D(Gi)-mCherry injected in the POA of Fos-2A-iCreER mice treated with light and 4-Hydroxytamoxifen did not show mCherry expression in somas of the SCN (**a**), or the SON (**b**). n = 6 animals. Scale bars denote 200 μm. SCN, suprachiasmatic nucleus; SON, supraoptic nucleus; POA, preoptic area.

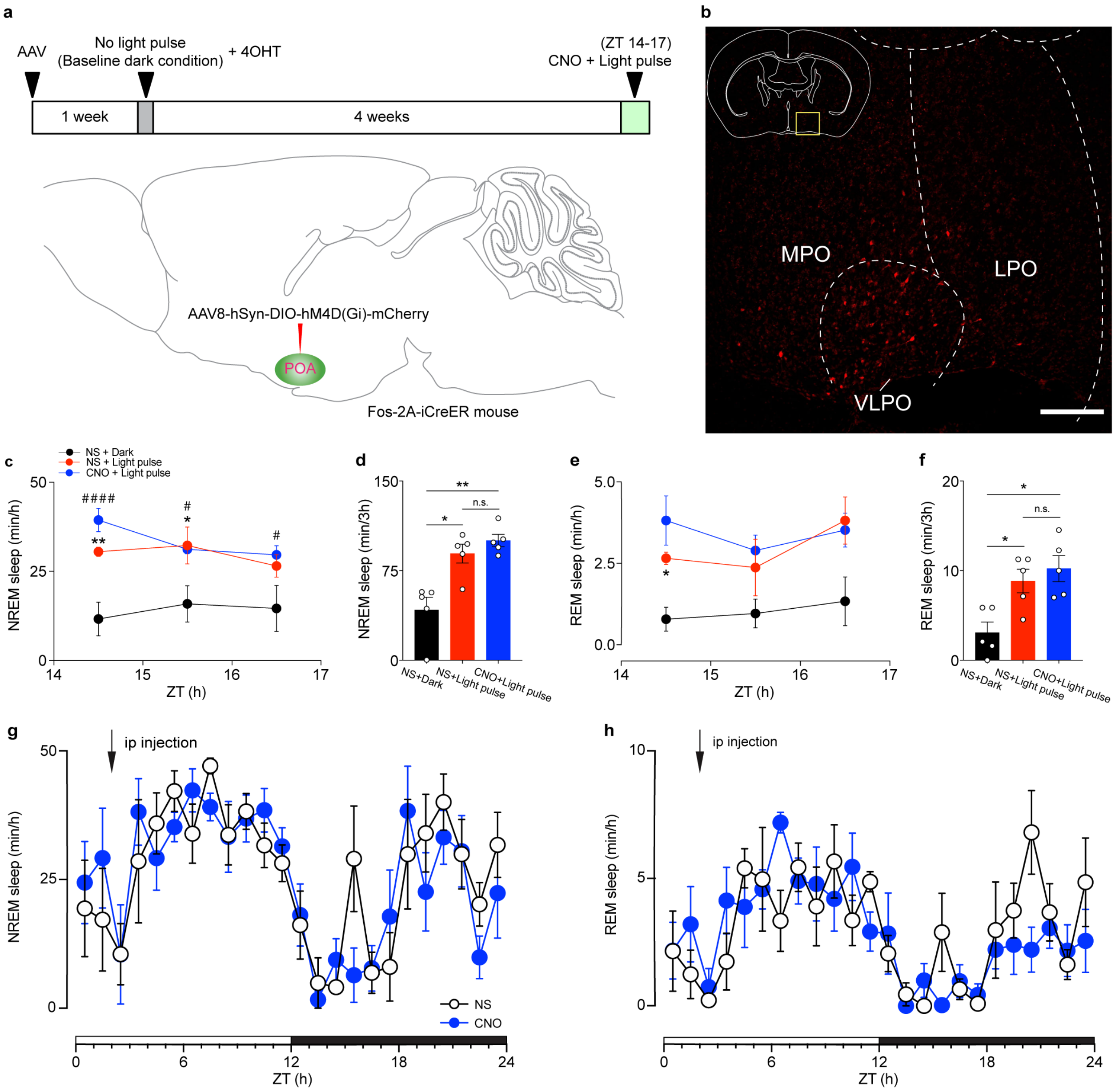

Supplementary Figure 9 Inhibiting night-active POA neurons does not influence sleep. **a,** Schematic showing viral, 4-Hydroxytamoxifen (4-OHT) and clozapine-N-oxide (CNO) injections in Fos-2A-iCreER mice. **b,** 4-OHT induced expression of mCherry in night-active POA neurons (n = 5 animals; scale bar, 200 µm). **c,** Time course changes in NREM sleep after normal saline (NS) or CNO injection during light pulse from zeitgeber time (ZT) 14 to ZT 17 (n = 5 animals; two-way ANOVA, $F_{2, 12} = 14.00$, $P = 0.0007$, Bonferroni post hoc test. $^{*}P < 0.05$, $^{*}P < 0.01$ indicates significant difference between NS+Light pulse and NS+Dark. $^{\#}P < 0.05$, $^{\#\#\#\#}P < 0.0001$ indicates significant difference between CNO+Light pulse and NS+Dark.). Each

cycle represents the hourly mean ± SEM of sleep. **d,** Total time in NREM sleep for 3 h after NS or CNO injection (n = 5 animals; one-way ANOVA, $F_{1.608, 6.432} = 20.81$, $P = 0.0019$, Bonferroni post hoc test, *$P < 0.05$, **$P < 0.01$). **e,** Time course changes in REM sleep after NS or CNO injection during light pulse from ZT 14 to ZT 17 (n = 5 animals; two-way ANOVA, $F_{2, 12} = 8.257$, $P = 0.0056$). Each cycle represents the hourly mean ± SEM of sleep. **f,** Total time in REM sleep for 3 h after NS or CNO injection (n = 5 animals; one-way ANOVA, $F_{2.000, 11.71} = 8.257$, $P = 0.0058$, Bonferroni post hoc test, *$P < 0.05$). **g,** Time course changes in NREM sleep after NS or CNO injection (n = 4 animals; two-way ANOVA, $F_{1, 6} = 0.2188$, $P = 0.6565$, Bonferroni post hoc test). Each cycle represents the hourly mean ± SEM of sleep. The white and black bars on the x axes indicate light-on and light-off periods, respectively. **h,** Time course changes in REM sleep after NS or CNO injection (n = 4 animals; two-way ANOVA, $F_{1, 6} = 0.2236$, $P = 0.6530$). All error bars denote SEM. MPO, medial preoptic area; LPO, lateral preoptic area; VLPO, ventrolateral preoptic nucleus.

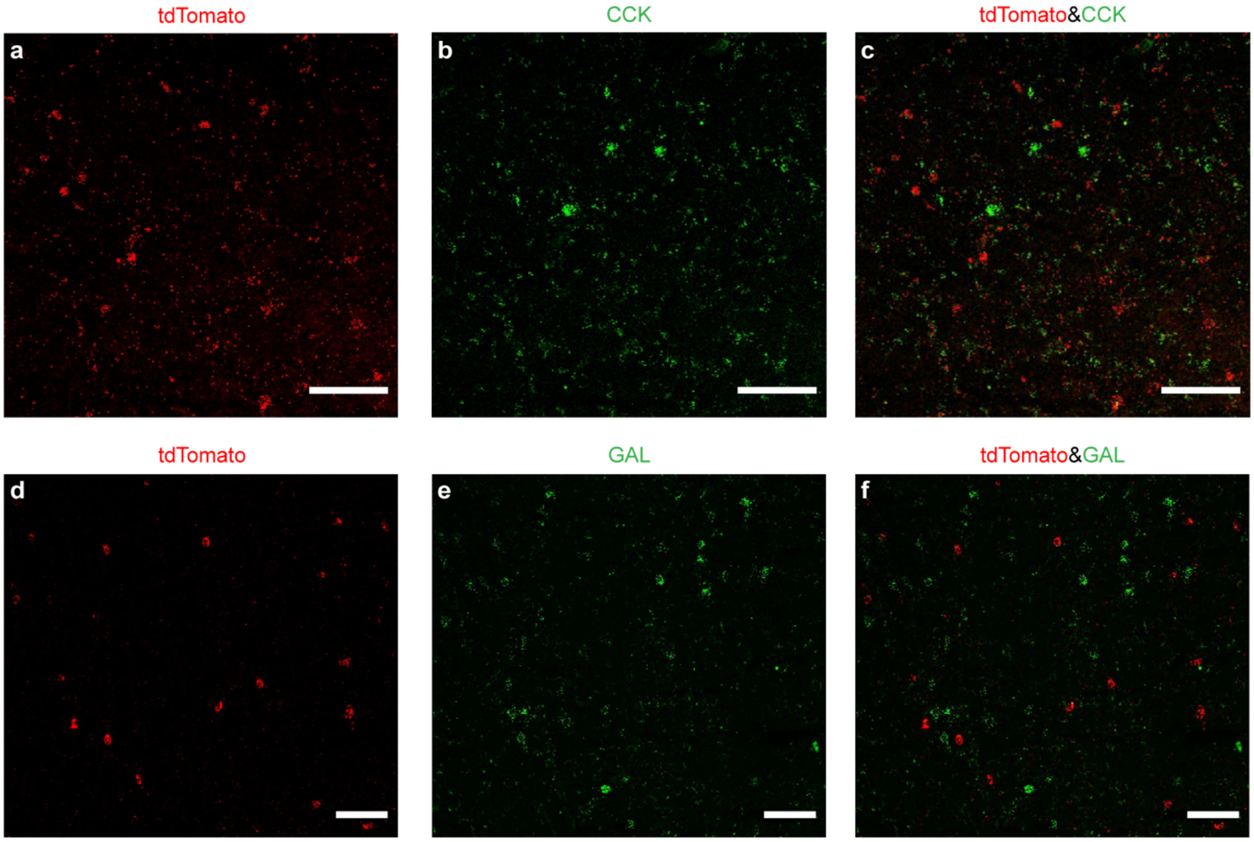

Supplementary Figure 10 Characterization of light-responsive neurons in the POA. **a-c,** Distributions of tdTomato positive neurons (**a**), CCK positive neurons (**b**), and colocalization (**c**) in the POA. **d-f,** Distributions of tdTomato positive neurons (**d**), GAL positive neurons (**e**), and colocalization (**f**) in the POA. Note that both CCK and GAL are not co-labeled with tdTomato. n = 4 animals. Scale bars denote 100 μm. CCK, cholecystokinin; GAL, galanin.

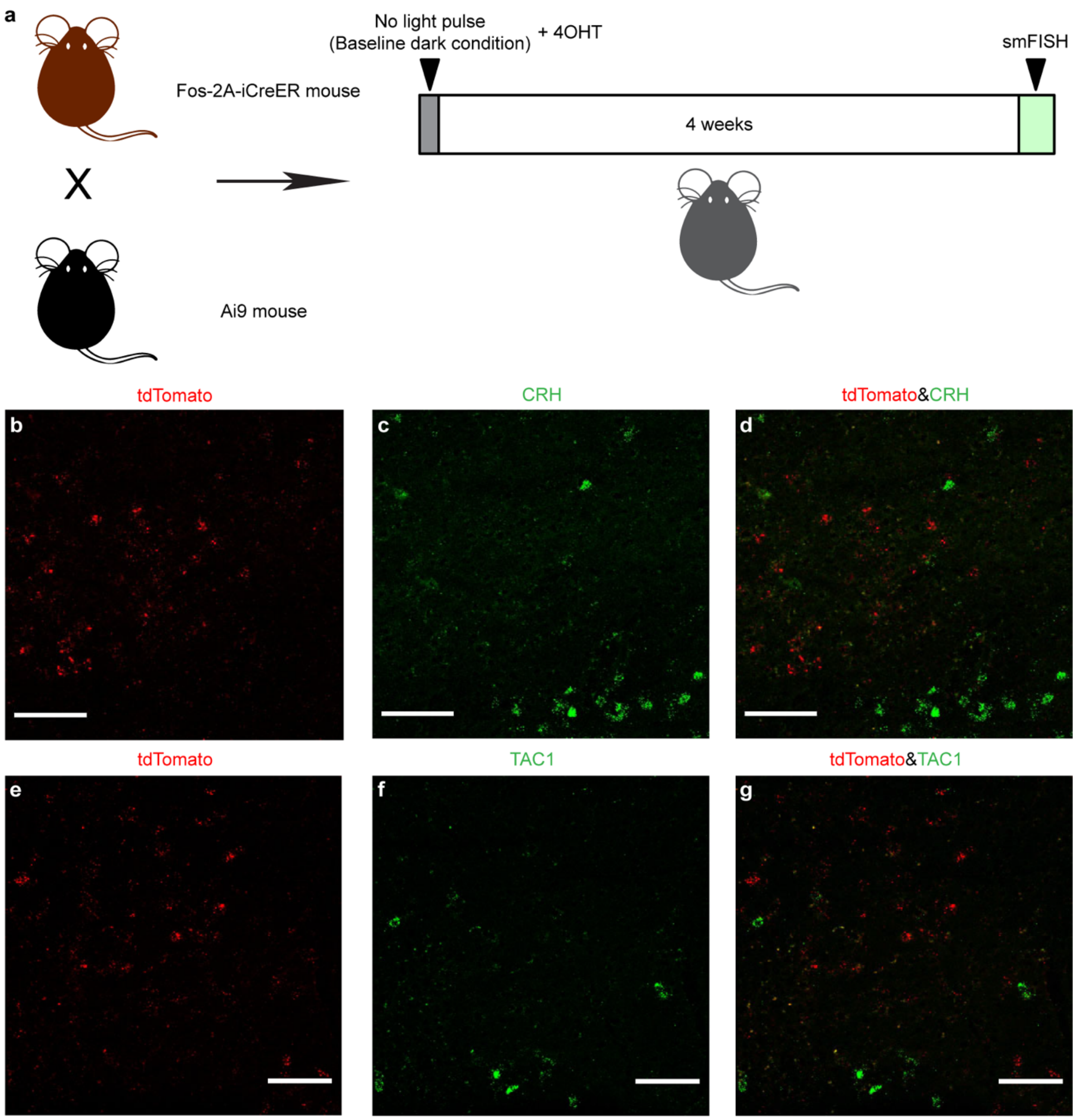

Supplementary Figure 11 Characterization of night-active neurons in the POA. **a,** Schematic showing Fos-2A-iCreER and CAG-FLEX-tdTomato (Ai9) mice maintained in baseline dark condition, 4-Hydroxytamoxifen (4-OHT) injection and single-molecule fluorescence in situ hybridization (smFISH). **b-d,** Distributions of tdTomato positive neurons (**b**), CRH positive neurons (**c**), and colocalization (**d**) in the POA. **e-g,** Distributions of tdTomato positive neurons (**e**), TAC1 positive neurons (**f**), and colocalization (**g**) in the POA. Note that in contrast to Figure 4, there are no co-labeling of CRH and TAC1 neurons. n = 3 animals. Scale bars denote 100 μm. CRH, corticotropin-releasing hormone, TAC1, tachykinin-1.

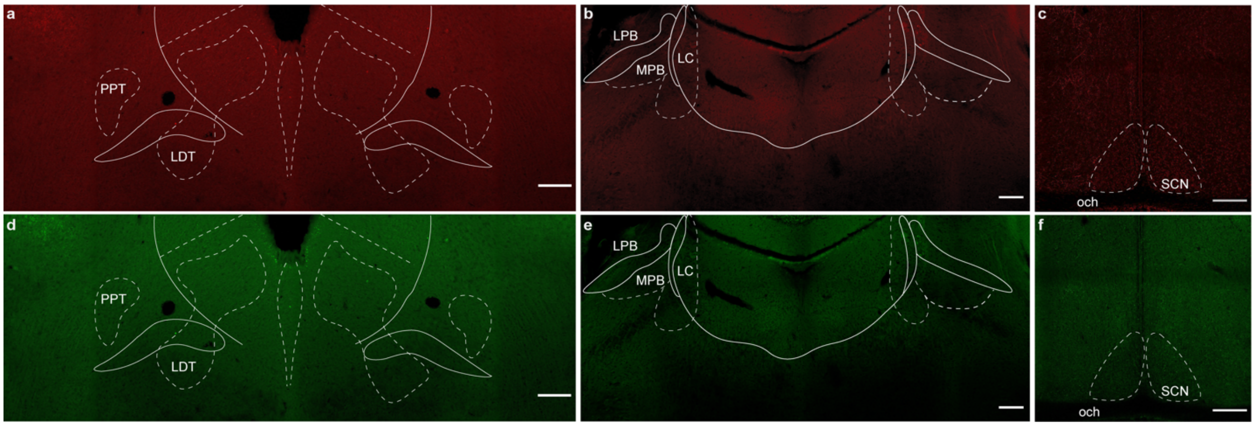

Supplementary Figure 12 POA light-responsive neurons do not project to the SCN, the PPT, the LDT, the LPB, the MPB or the LC. **a,** Distributions of tdTomato in the PPT and the LDT. **b,** Distributions of tdTomato in the LPB, the MPB and the LC. **c,** Distributions of tdTomato in the SCN. **d,** Distribution of EGFP in the PPT and the LDT. **e,** Distributions of EGFP in the LPB, the MPB and the LC. **f,** Distribution of EGFP in the SCN. PPT, pedunculopontine nucleus; LDT, laterodorsal tegmental nucleus; LPB, lateral parabrachial nucleus; MPB, medial parabrachial nucleus; LC, locus coeruleus; SCN, suprachiasmatic nucleus; och, optic chiasm. n = 3 animals. Scale bars denote 200 μm.
