# Supplementary material for: The retinal ipRGC-preoptic circuit mediates the acute effect of light on sleep
Source: Nat Commun. 2021 Aug 25;12:5115. doi: 10.1038/s41467-021-25378-w (PMC8387462; doi:10.1038/s41467-021-25378-w)
Supplement: Supplementary file 3 — Reporting Summary [file 41467_2021_25378_MOESM3_ESM.pdf]

nature research

Corresponding author(s): Ze Zhang, Samer Hattar

Last updated by author(s): Jul 21, 2021

Reporting Summary

Nature Research wishes to improve the reproducibility of the work that we publish. This form provides structure for consistency and transparency in reporting. For further information on Nature Research policies, see our Editorial Policies and the Editorial Policy Checklist.

Statistics

For all statistical analyses, confirm that the following items are present in the figure legend, table legend, main text, or Methods section.

n/a	Confirmed	
☐	☒	The exact sample size (*n*) for each experimental group/condition, given as a discrete number and unit of measurement
☐	☒	A statement on whether measurements were taken from distinct samples or whether the same sample was measured repeatedly
☐	☒	The statistical test(s) used AND whether they are one- or two-sided<br>*Only common tests should be described solely by name; describe more complex techniques in the Methods section.*
☒	☐	A description of all covariates tested
☐	☒	A description of any assumptions or corrections, such as tests of normality and adjustment for multiple comparisons
☐	☒	A full description of the statistical parameters including central tendency (e.g. means) or other basic estimates (e.g. regression coefficient) AND variation (e.g. standard deviation) or associated estimates of uncertainty (e.g. confidence intervals)
☐	☒	For null hypothesis testing, the test statistic (e.g. *F*, *t*, *r*) with confidence intervals, effect sizes, degrees of freedom and *P* value noted<br>*Give P values as exact values whenever suitable.*
☒	☐	For Bayesian analysis, information on the choice of priors and Markov chain Monte Carlo settings
☒	☐	For hierarchical and complex designs, identification of the appropriate level for tests and full reporting of outcomes
☐	☒	Estimates of effect sizes (e.g. Cohen's *d*, Pearson's *r*), indicating how they were calculated

Our web collection on statistics for biologists contains articles on many of the points above.

Software and code

Policy information about availability of computer code

Data collection	Ponemah software (DSI), version 6.41
Data analysis	NeuroScore (DSI), version 3.3.1<br>ImageJ software (NIH, USA), version 2.0.0-rc-69/1.52n<br>GraphPad Prism, version 8.0.2

For manuscripts utilizing custom algorithms or software that are central to the research but not yet described in published literature, software must be made available to editors and reviewers. We strongly encourage code deposition in a community repository (e.g. GitHub). See the Nature Research guidelines for submitting code & software for further information.

Data

Policy information about availability of data

All manuscripts must include a data availability statement. This statement should provide the following information, where applicable:

- Accession codes, unique identifiers, or web links for publicly available datasets
- A list of figures that have associated raw data
- A description of any restrictions on data availability

The sleep and histology data generated in this study are provided in the Supplementary Information/Source Data file.

Field-specific reporting

Please select the one below that is the best fit for your research. If you are not sure, read the appropriate sections before making your selection.

[x] Life sciences [ ] Behavioural & social sciences [ ] Ecological, evolutionary & environmental sciences

For a reference copy of the document with all sections, see nature.com/documents/nr-reporting-summary-flat.pdf

Life sciences study design

All studies must disclose on these points even when the disclosure is negative.

Sample size	No statistical methods were used to pre-determine the number of subjects in our study but our sample sizes are similar to those reported in previous publications (Danqian Liu et al., 2020 Science; Shinjae Chung et al., 2017 Nature; Zhe Zhang et al., 2019 Cell).
Data exclusions	AAV injection sites were confirmed post hoc by assessing reporter expression. Mice without correct targeting of tracers and/or vector were excluded from this study.
Replication	All experiments were repeated independently as indicated in the figure legends and the reproducibilities were confirmed.
Randomization	Mice were randomly assigned to control or experimental group.
Blinding	Experimenters were blinded to animals' identity for histological verification of viral expression location and thereby for the inclusion/exclusion of animals. Experimenters were also blinded to group allocation for sleep data analysis.

Reporting for specific materials, systems and methods

We require information from authors about some types of materials, experimental systems and methods used in many studies. Here, indicate whether each material, system or method listed is relevant to your study. If you are not sure if a list item applies to your research, read the appropriate section before selecting a response.

Materials & experimental systems

n/a	Involved in the study	
[ ]	[x]	Antibodies
[x]	[ ]	Eukaryotic cell lines
[x]	[ ]	Palaeontology and archaeology
[ ]	[x]	Animals and other organisms
[x]	[ ]	Human research participants
[x]	[ ]	Clinical data
[x]	[ ]	Dual use research of concern

Methods

n/a	Involved in the study	
[x]	[ ]	ChIP-seq
[x]	[ ]	Flow cytometry
[x]	[ ]	MRI-based neuroimaging

Antibodies

Antibodies used	chicken anti-GFP (ab13970, Abcam)<br>goat anti-RFP (LS-C340696, LSBio)<br>rabbit anti-OPN4 (AB-N38, Advanced Target Systems)
Validation	Specificity of the chicken anti-GFP antibody was validated by the manufacturer using immunofluorescence, immunohistochemistry, and western blot (www.abcam.com/gfp-antibody-ab13970.html) and previous publication (Britta Schürmann et al., 2020 Mol Psychiatry).<br>Specificity of the goat anti-RFP antibody was validated by the manufacturer using immunofluorescence and western blot (https://www.lsbio.com/antibodies/tdtomato-antibody-if-immunofluorescence-ihc-wb-western-ls-c340696/351334) and previous publication (Stephanie L Tsai et al., 2020 eLife).<br>Specificity of the goat anti-RFP antibody was validated by the manufacturer using immunofluorescence (https://atsbio.com/products/abn38/) and previous publication (Maureen E. Stabio et al., 2018 Neuron).

Animals and other organisms

Policy information about studies involving animals; ARRIVE guidelines recommended for reporting animal research

Laboratory animals	Fos-2A-iCreER (Stock # 030323) and CAG-FLEX-tdTomato (Ai9) (Stock # 007909) mice were obtained from the Jackson Laboratory. Opn4 Cre mice used in this study were obtained by mating the previous Opn4 Cre mice that were generated by our lab using targeting arms and general strategy (Jennifer L. Ecker et al., 2010 Neuron). Wild type mice (Stock # 101043) were obtained from the Jackson Laboratory.

	6-8 weeks old male mice were used at the start of the experimental procedures.<br>Mice had access to food and water ad libitum and were maintained at constant ambient temperature (21-23 °C), humidity (40-60%), and 12 h light/dark cycle (100 lux, light on at 06:00 am)
Wild animals	This study did not involve wild animals.
Field-collected samples	This study did not involve samples collected from the field.
Ethics oversight	All animal care and experimental procedures were approved by the Animal Care and Use Committees of the National Institute of Mental Health.

Note that full information on the approval of the study protocol must also be provided in the manuscript.
